# Common Repeat Elements in the Mitochondrial and Plastid Genomes of Green Algae

**DOI:** 10.3389/fgene.2020.00465

**Published:** 2020-05-12

**Authors:** David Roy Smith

**Affiliations:** Department of Biology, University of Western Ontario, London, ON, Canada

**Keywords:** *Chlamydomonas*, chloroplast DNA, genome size, *Haematococcus*, inverted repeat, mitochondrial DNA, palindrome, *Stephanosphaera*

## Abstract

Despite both originating from endosymbiotic bacteria, one does not typically expect mitochondrial DNA (mtDNA) to show strong sequence identity to plastid DNA (ptDNA). Nevertheless, a recent analysis of *Haematococcus lacustris* revealed exactly that. A common repeat element has proliferated throughout the mtDNA and ptDNA of this chlamydomonadalean green alga, resulting in the unprecedented situation whereby these two distinct organelle genomes are largely made up of nearly identical sequences. In this short update to the work on *H. lacustris*, I highlight another chlamydomonadalean species (*Stephanosphaera pluvialis*) for which matching repeats have spread throughout its organelle genomes (but to a lesser degree than in *H. lacustris*). What’s more, the organelle repeats from *S. pluvialis* are similar to those from *H. lacustris*, suggesting that they have a shared origin, and perhaps existed in the mtDNA and ptDNA of the most recent common ancestor of these two species. However, my examination of organelle genomes from other close relatives of *H. lacustris* and *S. pluvialis* did not uncover further compelling examples of common organelle repeat elements, meaning that the evolutionary history of these repeats might be more complicated than initially thought.

## Introduction

Mitochondrial and plastid DNAs (mtDNAs and ptDNAs) are no strangers to repeats. In fact, the organelle genomes of many diverse species are distended with non-coding repetitive DNA, which can come in a wide range of forms, from short direct repeats to long complex ones (Figueroa-Martinez et al., 2017; Brázda et al., 2018; Čechová et al., 2018; Wynn and Christensen, 2019). The repeats within mtDNA and ptDNA often share certain similarities with each other, such as a propensity for AT or GC nucleotides, but their sequences are typically unique. This is why the recent discovery of nearly identical repeats in the mitochondrial and plastid genomes of the chlamydomonadalean green alga *Haematococcus lacustris*, strain UTEX 2505, was so remarkable (Zhang et al., 2019).

A common family of GC-rich repeat elements, many of which are palindromic (i.e., a sequences that can be folded into hairpin structures), have spread throughout the *H. lacustris* mtDNA and ptDNA, resulting in the unprecedented situation whereby these two different genomes are largely made up of matching sequences (Zhang et al., 2019). The proliferation of these elements has resulted in extremely high organelle GC compositions (∼50%) (Smith, 2012) as well as severe genome expansion—the plastome of *H. lacustris*, at 1.35 Mb, is the largest on record (Bauman et al., 2018; Smith, 2018) and its mtDNA (124.6 kb) is among the biggest from green algae (Zhang et al., 2019; Repetti et al., 2020).

Repeat elements, particularly palindromic ones, are rampant in the organelle genomes of other chlamydomonadalean algae (Smith and Lee, 2009; Del Vasto et al., 2015; Figueroa-Martinez et al., 2017). To the best of my knowledge, however, *H. lacustris* is the only known species, of all eukaryotes, for which the same repeat has proliferated in both the mtDNA and ptDNA, notwithstanding examples of small regions of similarity between mitochondrial and chloroplast genomes (Pombert et al., 2005; Wang et al., 2007; Turmel et al., 2016). Here, in this brief update to the earlier work on the *H. lacustris* organelle DNAs, I highlight another chlamydomonadalean species that harbors nearly identical repeats in its mitochondrial and plastid genomes and then use these data to further explore the origins of such a strange phenomenon.

## Results and Discussion

### Common Repeats in the Organelle Genomes of *Stephanosphaera*

During the initial characterization of the *H. lacustris* organelle genomes, it was noted that the mitochondrial and plastid repeats show similarity not only to each other but also to the GC-rich repeats in the ptDNA of another chlamydomonadalean: *Stephanosphaera pluvialis*, strain SAG 78-1a (Zhang et al., 2019). The plastid genome of this colonial fresh-water alga, which is a close relative of *H. lacustris* (Buchheim et al., 2013), was sequenced as part of a large-scale phylogenetic analysis (Lemieux et al., 2015), but it remains in a highly fragmented state (111 contigs; accumulative length 220.8 kb; overall GC content 46%), likely because the wealth of repeats prevented its accurate assembly. The sequence identity between the *H. lacustris* repeats and those in the *S. pluvialis* ptDNA raise the obvious question: are these same repeats also found in the *S. pluvialis* mtDNA?

Currently, there are no publicly available mtDNA sequence data for *S. pluvialis*. However, this alga (specifically, the strain used for plastome sequencing, SAG 78-1a) was one of many species to have its transcriptome sequenced as part of the One Thousand Plant Transcriptomes Initiative (2019). RNA-sequencing (RNA-seq) data have proven to be an excellent resource for mining organelle transcripts from green algae (Sanitá Lima and Smith, 2017) and have even been used to reconstruct complete chlamydomonadalean organelle genomes (Tian and Smith, 2016). By downloading the *S. pluvialis* transcriptome and searching it via BLAST using chlamydomonadalean mitochondrial genes as queries, I was able to identify 18 contigs corresponding to putative mtDNA-derived transcripts (Table 1). These mitochondrial contigs range from 109 to 1,946 nt (avgerage length = 627 nt), have an accumulative length of 11,293 nt, and together contain the standard cohort of genes typically found in chlamydomonadalean mitochondrial genomes, including fragmented and scrambled rRNAs (Table 1). Half of the contigs appear to have mitochondrial introns and most include sections of transcribed intergenic DNA, providing 5,705 nt of non-coding sequence data to investigate the presence/absence of repeat elements.

**TABLE 1:** Mitochondrial RNA-derived contigs identified from the *Stephanosphaera pluvialis* SAG 78-1a One Thousand Plant transcriptome data.

**Contig name**	**Length (nt)^1^**	**Gene^2^**	**Putative intron^3^**	**Non-coding length (nt)^4^**	**Shared repeats with ptDNA (length nt)**
ZLQE-2001336	1946	*rrnS2*	Yes	1335	Yes (160)
ZLQE-2001334	1454	*cox1*	Yes	302	Yes (70)
ZLQE-2027293	1252	*cob*	No	22	No
ZLQE-2001337	1108	*rrnL5*	No	880	Yes (180)
ZLQE-2001335	952	*cox1*	Yes	595	Yes (75)
ZLQE-2004969	716	*rrnL3*	No	563	No
ZLQE-2001333	659	*cox1*	Yes	413	No
ZLQE-2022777	493	*rrnL7*	No	321	No
ZLQE-2023888	482	*rrnL6*	No	204	Yes (50)
ZLQE-2022382	406	*nad2*	Yes	171	No
ZLQE-2020255	273	*rrnL2*	Yes	197	Yes (50)
ZLQE-2019931	263	*nad6*	No	71	No
ZLQE-2016101	254	*rrnS4*	No	185	Yes (50)
ZLQE-2019351	248	*rrnL4*	No	148	Yes (30)
ZLQE-2018739	230	*nad2*	Yes	125	No
ZLQE-2018559	226	*rrnL2*	Yes	61	No
ZLQE-2018441	222	*nad4*	Yes	108	Yes (40)
ZLQE-2010979	109	*nad5*	No	4	No

*^1^In some cases lengths are slightly longer than One Thousand Plant data due to polishing and extension of contigs. ^2^In many cases, contigs contain only partial coding regions. ^3^Any interruptions in coding sequences >50 nt were considered to be putative introns. ^4^Includes intergenic and intronic sequences.*

**FIGURE 1:**
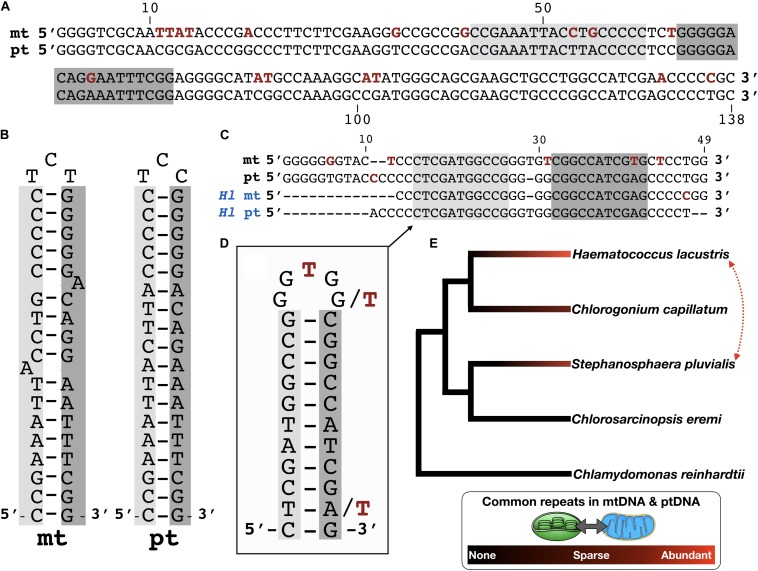
Common repeats in the organelle genomes of *Stephanosphaera pluvialis*. **(A)** Pairwise nucleotide alignment of a shared repeat element in the *S. pluvialis* mitochondrial (mt) and chloroplast (pt) genomes. Polymorphisms are highlighted in red. Palindromic repeat highlighted in light and dark gray. Region corresponds to nucleotides 95–232 of accession KT625314 and nucleotides 558–695 from contig ZLQE-2001337. Note, other smaller palindromes are also found in these sequences but are not shown. **(B)** Folded hairpin structure of the palindromic repeat shown in panel **(A)**. **(C)** Pairwise alignment of a shared repeat element in the mitochondrial (mt) and chloroplast (pt) genomes of *S. pluvialis* and those of *Haematococcus lacustris* (*Hl* mt and *Hl* pt). Palindromic repeat highlighted in light and dark gray. **(D)** Folded hairpin structure of palindrome shown in panel **(C)**. **(E)** Common repeats in the mitochondrial and chloroplast genomes of green algae. Branching order based on Fučíková et al. (2019). Dotted red line denotes similar repeats in different species.

Sure enough, nine of the *S. pluvialis* mitochondrial transcripts contain repeats, including palindromes, that match to those from the neighboring ptDNA with ≥80% sequence identity (Table 1 and Figure 1A). In total, ∼700 nt of the mitochondrial contigs can be aligned to ptDNA repeats, meaning that the *S. pluvialis* organelle genomes harbor nearly identical repeat elements. These elements were identified by blasting the 18 mitochondrial contigs against a database made up of the *S. pluvialis* ptDNA (GenBank accessions KT625299-KT625409). As with *H. lacustris*, the *S. pluvialis* organelle repeats are GC-rich (>50%); moreover, a single repeat from the mitochondrial genome can match to hundreds of locations in the plastid genome, and palindromes were found in both intronic and intergenic regions. However, unlike *H. lacustris*, the segments of the *S. pluvialis* mitochondrial contigs that show similarity to ptDNA are relatively short (approximately 30–180 nt) and encompass only a small proportion (∼12%) of the analyzed regions (Table 1). The same cannot be said for the repeats in the *S. pluvialis* plastome, which appear to be widespread throughout much of the sequenced non-coding ptDNA (Lemieux et al., 2015). But keep in mind that these observations (which are intended to help direct future research) are based on partial mitogenome and plastome data and will need to be revised upon complete organelle genome sequencing of *S. pluvialis*.

### The Mitochondrial Palindromic Repeats Are in Disrepair

Close inspection of the matching sequences between the *S. pluvialis* organelle genomes reveals an interesting trend: the palindromes from the mitochondrial genome typically contain more imperfections than their ptDNA counterparts (Figures 1A–D). This point can be easily interpreted by comparing the folded hairpin structures of mitochondrial palindromes to those from the plastome (Figures 1B,D). For example, the palindromes from the mitochondrial genome often contain mismatches and/or insertion-deletion mutations in the stem portion of the hairpin, which is not necessarily true for the corresponding ptDNA palindromes (Figures 1B,D). These imperfections could be an indication that the mitochondrial palindromic repeats are in a state of deterioration, or at least are not being as well maintained as those in the ptDNA. If this hypothesis is correct, it could explain why the palindromes are more widespread within the *S. pluvialis* ptDNA and might also indicate that they appeared first in the plastome and spread via intracellular DNA transfer to the mtDNA (Smith, 2011)—but see discussion below. Or it could just signal that the mtDNA has a higher rate of silent-site nucleotide substitution than the ptDNA, which is a common theme among eukaryotic algae (Smith, 2015). Keep in mind as well that GC-rich palindromic repeats are thought be transposable elements in certain organelle genomes (Wu and Hao, 2015).

Even more intriguing is the resemblance of the *S. pluvialis* mitochondrial and plastid repeats with those from *H. lacustris* (Figures 1C,D). Indeed, the organelle repeats from these two distinct species can share moderately strong sequence identity (>80%) with each other over regions that can exceed 100 nt. For *S. pluvialis*, however, the palindromes in the ptDNA show stronger similarity to those in the *H. lacustris* organelle genomes than the mitochondrial ones do (Figures 1C,D), further supporting the idea that the *S. pluvialis* mitochondrial palindromes are in disrepair. Nevertheless, these observations imply that the organelle DNA palindromes in *S. pluvialis* and *H. lacustris* have a shared origin, and perhaps existed in the mtDNA and ptDNA of the most recent common ancestor of these two species. To investigate this idea further, I explored the organelle genomes of close relatives of *S. pluvialis* and *H. lacustris* for palindromic elements.

### Palindromic Repeats in Other Species

The phylogenetic relationships among chlamydomonadalean algae are reasonably well resolved (Nakada et al., 2008a; Lemieux et al., 2015; Fučíková et al., 2019), including for species from the Chlorogonia and Stephanosphaerinia, the respective clades to which *H. lacustris* and *S. pluvialis* belong (Buchheim et al., 2013; Pegg et al., 2015). I cross-referenced members of these two clades against available organelle genome sequences, which, in turn, allowed me to investigate the mtDNA and ptDNA of an additional two species closely affiliated to *H. lacustris* and *S. pluvialis* for common organelle repeat elements (Figure 1E).

One of the closest known relatives of *H. lacustris* is the unicellular freshwater alga *Chlorogonium capillatum* (Nakada et al., 2010; Buchheim et al., 2013). This species has had its mitochondrial genome completely sequenced (Kroymann and Zetsche, 1998) and there is a nearly complete assembly of its ptDNA (Lemieux et al., 2015); these data come from two distinct but very closely related strains of *C. capillatum*: SAG 12-2e (mtDNA) and UTEX 11 (ptDNA) (Nozaki et al., 1998; Nakada et al., 2008b). [Note: SAG 12-2e was previously referred to as *Chlorogonium elongatum*.] The *C. capillatum* mitogenome is not particularly big or bloated (22.7 kb; ∼47% non-coding DNA), but it does contain repeats, including palindromic ones (Kroymann and Zetsche, 1998). The plastome, on the other hand, is large and expanded (>271 kb; >64% non-coding) and it, too, contains palindromic repeats, significantly more than the mtDNA. But are the mitochondrial and plastid repeats similar to one another?

Comparison of the *C. capillatum* organelle genomes using BLAST did uncover some regions of microhomology within non-coding regions. For example, two ∼60 nt segments from the mitogenome each match to a distinct location in the plastome with ∼75% sequence identity. Both of these segments correspond to GC-rich repeats (Kroymann and Zetsche, 1998), part of which can be folded into hairpin-like secondary structures. There were also dozens of short (20–30 nt) mtDNA regions showing high pairwise identity (90–95%) to the ptDNA, some of which represent palindromic elements. Thus, these two genomes do have some common repeats, but not to the same high degree as found in the organelle DNAs of *H. lacustris* and *S. pluvialis*. Moreover, the *C. capillatum* repeats show no obvious sequence similarity to those of the latter two species, which is surprising given that *C. capillatum* is believed to share a common ancestor with *H. lacustris* more recently than it does with *S. pluvialis* (Nakada et al., 2010; Buchheim et al., 2013).

I was also able to explore a close relative of *S. pluvialis* for common organelle repeat elements, namely *Chlorosarcinopsis eremi* strain MKA.28 (Figure 1E). This unicellular freshwater alga, which is normally found in desert environments (Juy-abad et al., 2018), has recently had its mitogenome and plastome completely sequenced (Fučíková et al., 2019; Juy-abad et al., 2019). The ptDNA is expanded (∼298 kb; ∼67% non-coding) and populated with hundreds of short palindromic repeats, which have been described in detail and do not show high sequence identity with those from other chlamydomonadalean species (Smith, 2020). Conversely, the mtDNA is small (24.9 kb) and essentially devoid of palindromes (Juy-abad et al., 2019; Smith, 2020). Nevertheless, I compared these two genomes using BLAST to see if any of the ptDNA palindromes matched to the mitogenome. Apart from short similarities among coding regions (e.g., a plastid rRNA gene matching to a mitochondrial one), the *C. eremi* organelle genomes are almost entirely made up of distinct non-coding sequences. This, again, is surprising given that *C. eremi* and *S. pluvialis* are more closely related to each other than to *H. lacustris*.

## Conclusion

After all this, I feel like I am no further ahead in understanding how a common repeat element has proliferated throughout the mitochondrial and plastid genomes of *H. lacustris* and *S. pluvialis*. These data still leave open—but do not completely support—the scenario that the common ancestor of the Chlorogonia and Stephanosphaerinia clades had matching palindromes in its mtDNA and ptDNA and that these repeats have been preserved for millions of years. Perhaps more plausible is that the shared palindromes in these two species owe their origin to horizontal DNA transfer, both between species and between organelles within a cell. Precisely how this occurs is debated, but the lateral movement of DNA between distinct organelle genomes is well documented (Bergthorsson et al., 2003; Stegemann et al., 2012), particularly intracellular plastid-to-mitochondrion DNA transfer, which is especially prevalent in species with multiple plastids per cell (Smith, 2011). *H. lacustris* and *S. pluvialis*, however, have a single plastid per cell (Ettl, 1983; Nakada and Ota, 2016), which should greatly reduce the potential for ptDNA-to-mtDNA transmission. Their mitochondria, on the other hand, can apparently exist in multiple numbers per cell (Wayama et al., 2013), which should increase the probably of successful mtDNA-to-ptDNA transfers (Smith et al., 2011), contradicting my earlier suggestion above that the transfer might have occurred via ptDNA to mtDNA. Organelle introns are known to move between species and between mitochondria and plastids (Pombert et al., 2005), so it is possible that the repeats piggybacked on a mobile intron. In this context, it is noteworthy that all of the organelle genomes discussed here contain introns, and the mtDNA and ptDNA introns from *H. lacustris* and *S. pluvialis* do harbor palindromes. Complete organelle DNA sequences (including the intronic regions) from *S. pluvialis* might provide better insights into this hypothesis.

If anything, these data reinforce the notion that mitochondrial and plastid genomes can have similar repeat sequences. However, *H. lacustris* still stands out as an exceptionally extreme case of a common repeat expansion in two distinct compartments. It will be interesting to see if the organelle genomes of even closer relative of *H. lacustris*, such as *Ettlia carotinosa* (Buchheim et al., 2013), and other species of *Haematococcus* comprise shared palindromic repeats. Finally, the presence/absence of palindromes within an organelle genome may seem trivial from a broad biological perspective, but recent analyses have shown that these types of sequences can have significant impacts on the evolution of organelle DNAs (Smith, 2020) and, thus, should not be overlooked.

## Methods

The One Thousand Plant Transcriptomes assembly for *S. pluvialis* can be found under accession number ZLQE; see Carpenter et al. (2019) for detailed instructions on accessing the data. Mitochondrial RNA-derived contigs were identified by blasting the *C. eremi* and *H. lacustris* mtDNAs (GenBank accessions NC_041430.1 and MK878592.1) against the *S. pluvialis* transcriptome with BlastN implemented through Geneious v10.2.6. (Biomatters Ltd., Auckland, New Zealand) using default settings. (Note: all other blast analyses described in the article were carried out using these same settings). Hits containing *bona fide* mitochondrial genes (Table 1) were polished by mapping the raw *S. pluvialis* RNA-seq data (GenBank accession ERX2100118) to the contigs using the Geneious read mapper (medium-low sensitivity; default settings); in a few instances, this resulted in minor (10–45 nt) extensions to the contigs.

## Data Availability Statement

The datasets used for this study can be found in The One Thousand Plant Transcriptomes assembly for *S. pluvialis* under accession number ZLQE.

## Author Contributions

DS wrote the manuscript and analyzed the data.

## Conflict of Interest

The author declares that the research was conducted in the absence of any commercial or financial relationships that could be construed as a potential conflict of interest.
